# The timecourse of space- and object-based attentional prioritization with varying degrees of certainty

**DOI:** 10.3389/fnint.2013.00088

**Published:** 2013-12-05

**Authors:** Leslie Drummond, Sarah Shomstein

**Affiliations:** Department of Psychology, George Washington UniversityWashington, DC, USA

**Keywords:** object-based attention, space-based attention, dynamic displays, attentional allocation, inhibition of return

## Abstract

The relative contributions of objects (i.e., object-based) and underlying spatial (i.e., space-based representations) to attentional prioritization and selection remain unclear. In most experimental circumstances, the two representations overlap thus their respective contributions cannot be evaluated. Here, a dynamic version of the two-rectangle paradigm allowed for a successful de-coupling of spatial and object representations. Space-based (cued spatial location), cued end of the object, and object-based (locations within the cued object) effects were sampled at several timepoints following the cue with high or low certainty as to target location. In the high uncertainty condition spatial benefits prevailed throughout most of the timecourse, as evidenced by facilitatory and inhibitory effects. Additionally, the cued end of the object, rather than a whole object, received the attentional benefit. When target location was predictable (low uncertainty manipulation), only probabilities guided selection (i.e., evidence by a benefit for the statistically biased location). These results suggest that with high spatial uncertainty, all available information present within the stimulus display is used for the purposes of attentional selection (e.g., spatial locations, cued end of the object) albeit to varying degrees and at different time points. However, as certainty increases, only spatial certainty guides selection (i.e., object ends and whole objects are filtered out). Taken together, these results further elucidate the contributing role of space- and object-representations to attentional guidance.

Attentional selection determines what subset of the sensory stimuli will be processed from the large amount of information available in the environment. Selection is based on at least two non-mutually exclusive representations: space- and object-based. The space-based representation is defined as the spatial coordinates of a target relative to the observer (or another reference point), while the object-based representation is defined by the surfaces that occupy those spatial coordinates. The individual contributions of these two representations to attentional guidance have been investigated extensively, but because objects occupy spatial locations, *true* separation is difficult to achieve and thus the individual contribution of each representation to selection remains elusive.

Much of the previous research on object-based attention has been conducted with the two-rectangle paradigm originally introduced by Egly et al. (1994). In this paradigm, two rectangles are aligned to create a perfect square. One end of one of the rectangles is cued, either by a luminance change, an overlapping color, or a shape. A target then appears either at the cued location (valid), the non-cued location at the opposite end of the cued object (same object, SO), or in the part of the non-cued object directly across from the cued location (different object, DO). There are two effects that arise as a result: (1) space-based or validity: targets that appear at the cued location are detected faster than those that appear in any other location (effect size of about 100 ms); and (2) object-based: the SO location targets are detected faster than the DO location targets (effect size of about 20 ms). The object-based difference is not a result of: (a) simple distance differences because the spatial separation from the cued location to possible locations on the same and different objects is identical, or (b) the object boundaries because the effect is seen with displays that utilize an occluder (Moore et al., 1998).

While the existence of the object-based effect is well established, several conflicting accounts of the precise factors required to observe the effect arose in the literature, and therefore the mechanism that gives rise to these effects remains under investigation. Originally, object-based effects were attributed to *attentional spreading *or the* grouped array principle *(Vecera and Farah, 1994; Avrahami, 1999; Shomstein, 2012). According to this view, attention is initially drawn to the cue, and then automatically spreads at a predictable rate down the cued object, resulting in a benefit for same-object over different-object targets. However, this hypothesis fails to explain circumstances under which object-based effects are *not* present. If attentional spreading is automatic, starting at the cued location and spreading to other locations within an object, then object-based effects should be ubiquitous. At a theoretical level, allocating a limited resource such as attention should be a maximally efficient process, and there are many circumstances in which a predictable spread based on salient but ultimately irrelevant cue is not the optimal strategy (e.g., when the target seldom appears in the cued object). *Attentional spreading* or the *grouped array principle* places many constraints on selection and suggests attentional allocation is rather limited and inflexible. Two recent, more flexible, mechanistic accounts have been put forth as alternatives in an effort to capture a full gamut of behaviors related to object-based attentional guidance.

*The attentional shifting *hypothesis, proposed by Lamy and Egeth (2002), argues that a mere shift of attention is sufficient to elicit object-based effects. In a series of several experiments, Lamy and Egeth (2002) demonstrated that whenever the task required shifts of attention, object-based effects were in fact observed. However, there is another variable that could potentially predict the presence of the object-based effect: positional uncertainty of the upcoming target. Shomstein and Yantis (2002) proposed the *attentional prioritization* hypothesis, which argues that with a high degree of uncertainty about spatial position of the upcoming target, and all things being equal, the highest attentional priority is assigned to the cued location and the same-object location, due to the consequence of lower level figure-ground segmentation processes (Kramer and Jacobson, 1991; Baylis and Driver, 1995; Watson and Kramer, 1999; Kimchi and Peterson, 2008; Shomstein, 2012). However, if spatial uncertainty is reduced (e.g., information is given about the location of the upcoming target), attentional allocation will be influenced by positional certainty, such that the highest priority will be allocated to the most likely target location. Under these circumstances, attention will no longer be guided by object representations.

A series of papers contributed evidence for *attentional prioritization *(Shomstein and Yantis, 2002, 2004; Chan and Hayward, 2008; Feria, 2008, 2010; Shomstein and Behrmann, 2008; Drummond and Shomstein, 2010; Lee and Shomstein, 2013; Shomstein and Johnson, 2013) by creating a set of circumstances in which the cue is either uninformative (i.e., uncertainty) or informative (i.e., certainty) of the location of the upcoming target. When the cue is uninformative, all possible target locations are equally likely to contain the target. Under these circumstances, all available information in the display is used to prioritize and constrain attentional guidance, including object representations. Highest priority is assigned to the valid (cued) location because the only information available is the sensory event (i.e., the cue) at that location. Next highest priority is given to the non-cued portion of the same object via figure-ground segmentation, followed by locations on the different (non-cued) object.

The *attentional prioritization* hypothesis, then, rests on results obtained under conditions of certainty. If information about the cue to target relationship is available (such as probabilities, or as in the case of Drummond and Shomstein (2010), an explicit rule describing the target’s upcoming location), attentional priority is biased toward the location that is most likely to contain the target. Certainty allows for a more efficient selection of the target either via the mechanism of an attentional saliency map or through a narrowing of attentional focus (Goldsmith and Yeari, 2003; Gottlieb et al., 2009a,b; Ipata et al., 2009), thereby eliminating object-based effects. Object representations do not contribute to attentional guidance presumably because they are effectively filtered out (Lee and Shomstein, 2013; Shomstein and Johnson, 2013). Selection is instead determined based on prior knowledge of the cue-to-target relationship and the fastest responses occur to the likely target location (second only to the cued location).

Framing space-based and object-based effects within the context of a priority map is supported by a growing body of research that has implicated several regions within the frontal and parietal cortex as the areas where such priority maps are established. In particular, single-unit physiology experiments with awake behaving monkeys have found evidence that the frontal eye fields (FEFs) and the lateral intraparietal area (LIP) contain representations compatible with priority maps (Platt and Glimcher, 1999; Thompson et al., 2005; Gottlieb et al., 2009a,b; Ipata et al., 2009). Concordantly, functional imaging studies in humans have found that corresponding frontal and parietal areas contain topographic representations related to saccade planning and attention (Molenberghs et al., 2007; Serences and Yantis, 2007; Silver and Kastner, 2009).

Several questions remain about the specifics of prioritization, most notably regarding the extent to which the space and object representations are prioritized. It is possible that spatial locations are prioritized exclusively or are prioritized to a greater extent than the object ends, or vice versa. It could also be the case that both representations are prioritized (and thus contribute to attentional guidance) to the same extent. In the original two-rectangle paradigm, or in any static paradigm, space and object-based representations overlap, as spatial coordinates are occupied by the object. Thus, a difference between cued and non-cued locations cannot be classified as purely a space- or object-based benefit. Generally, it is assumed that these effects are simply additive (Tipper et al., 1999), but it could also be the case that the overlap creates *over-*additive effects. However, in a dynamic display in which the objects move after the cue, it is possible to separate the space- and object-based representations to determine individual contributions to selection and the relationship between the two representations.

One of the many different ways of measuring attentional allocation is focusing on inhibition of return (IOR) in dynamic displays under conditions of uncertainty (Tipper et al., 1994, 1999; Becker and Egeth, 2000). The IOR effect is a cost (longer RTs) associated with a target appearing in the previously cued location, where enough time without the appearance of a target will repel attention toward other locations (Posner et al., 1985). Rather than constraining attention to the cued location indefinitely, as the effect of the sensory cue dissipates (around 700–900 ms), participants mark the cued location as “searched” and begin searching the rest of the display for the target. Therefore, IOR is a marker of the time it takes to return to the cued location after it has already been deemed unlikely to contain the target. The amount of inhibition observed is affected by task demands (i.e., how much load is on the attentional system), as well as how much interference is present in the display (Muller and Rabbitt, 1989; Wright and Richard, 2000; Lupianez et al., 2001; List and Robertson, 2007). Observing IOR effects for a location or an object, then, indicates that this particular location or object has been successfully selected by attention.

Previous studies have successfully separated space- and object-based representations in order to examine the extent to which objects ends contribute to attentional guidance. In a study by Becker and Egeth (2000), objects rotated out of their original locations after the cue, and thus were defined by their location at the end of the trial as follows: environment (cued spatial location), object (originally cued), across (directly opposite the cued spatial location), and adjacent (next to the cued spatial location). Targets appeared in each of the four locations equally often (i.e., high degree of uncertainty). Becker and Egeth (2000) found significant IOR effects for the *environment* and *object* conditions, replicating an earlier study by Tipper et al. (1994). Importantly, this result suggested that two representations are used concurrently and that they can be nested within one another (Tipper et al., 1999; Becker and Egeth, 2000; List and Robertson, 2007). These results were extended to objects with sub-regions, not unlike the two-rectangle paradigm of Egly et al. (1994), by using boomerang-shaped objects to determine if and how attention spreads across the display. The same four conditions were used and results demonstrated that IOR was also present on the non-cued portion of the object. Several other studies, including those using neuronal recording, suggest that activity in one representation (space or object) will initiate activity in the other and that multiple depths (or representations) can be selected at the same time (Moore and Fallah, 2001; Fallah et al., 2007). These results suggest not only that the two representations can be successfully separated, but that there are individual and distinct contributions of each to attentional guidance.

Thus far, we know that there are at least two representations within which attention is allocated (space and objects). The representations can be used concurrently (i.e., both effects can appear in the same experiment) and appear to be nested within each other (i.e., objects overlap spatial locations), a finding that is supported by neurophysiological evidence (Tipper et al., 1994, 1999; Muller and von Muhlenen, 1996; Moore and Fallah, 2001; Fallah et al., 2007). The timecourse of attention has been researched previously, but only with target location uncertainty (List and Robertson, 2007). All other previously mentioned studies maintained a constant inter-stimulus interval (ISI) and Stimulus Onset Asynchrony (SOA), while all possible locations were equally likely to contain the target (Tipper et al., 1994; Becker and Egeth, 2000). Therefore, the purpose of the experiments reported here is to directly investigate the role that underlying representations play in guiding attentional selection. Specifically we ask three questions: (a) which representation is being prioritized (spatial locations or object ends) and to what extent; (b) does the focus of attentional prioritization change over time; and most importantly, (c) how does the relative contribution of each representation change with various degrees of certainty?

## EXPERIMENT 1

**FIGURE 1:**
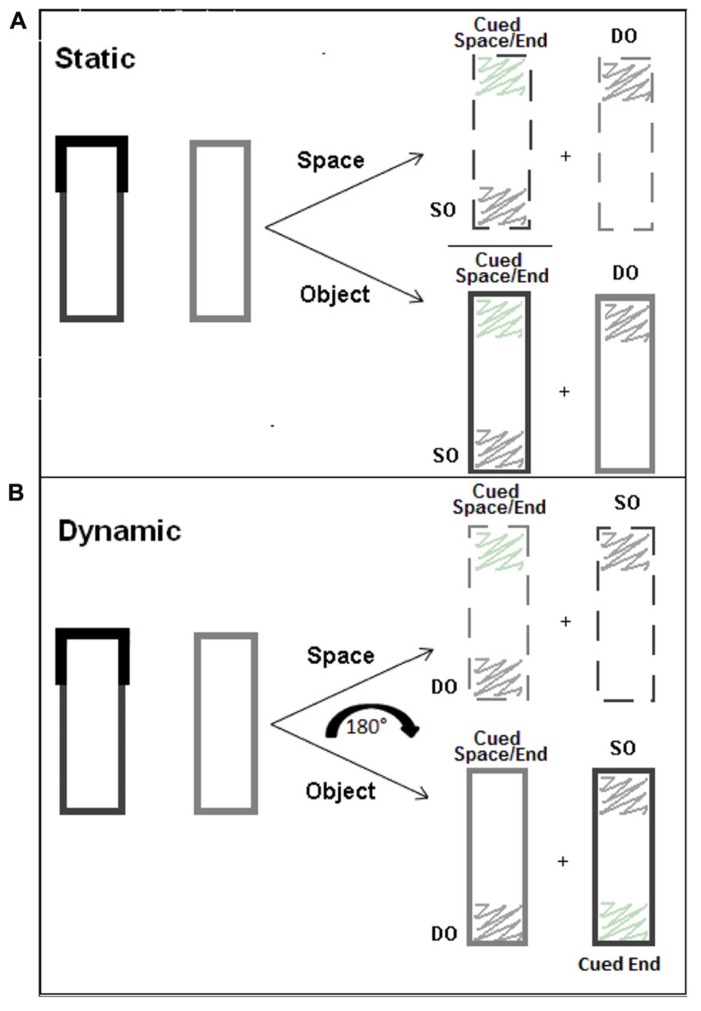
**Static (A) and dynamic **(B)** displays showing the overlap and separation of spatial and object representations (cued space, cued end of the object, same object [SO], different object [DO]).** In a static display, cued space and cued end are in the same spatial coordinates, whereas in a dynamic display, they are separated, which allows us to examine their separate attentional effects.

Experiment 1 uses a dynamic two-rectangle paradigm in order to elucidate individual contributions of object- and space-based representations to attentional guidance. Cuing a location and part of an object that occupies that spatial location and then rotating the objects will successfully separate the two attentional representations, space and object (see **Figure 1**). In a static display (**Figure 1A**), spatial locations are overlapped completely by the objects. Therefore, if a perceptual benefit for any particular location is observed it cannot be determined whether it arose from either a prioritized space- or object-based representation, or from a prioritization of both. In a dynamic display (**Figure 1B**), spatial locations and objects initially overlap, but once set in motion (180° rotation in the current study), the object ends separate from the underlying spatial coordinates (e.g., the end of the object that was cued ends the rotation diagonally from its starting point), and the two representations are no longer confounded. In this way, we can determine separate contributions for space and object representations.

The purpose of this experiment was to determine to what extent space- and object-based representations are prioritized by attentional selection under conditions of uncertainty, as well as to examine the temporal evolution of this selection. Participants had some knowledge about the upcoming target location based on the color of the cue. Blue cues indicated that the target would appear in the same spatial location, while red cues indicated the target had an equal chance of appearing in any of the three non-cued locations: cued end of the object; SO – location within the same object as the cue; DO – location equidistant to the cued part of the object but in a different object. To examine how attentional selection of multiple representations evolves over time, SOA between the cue and the upcoming target was manipulated. If only one representation is receiving the benefit of prioritization, we would expect only space-based, only cued end of the object, or only object cuing effects to occur across the time course. However, as previous studies indicate, representations could be used in tandem and their relative contributions could change as SOA increases (Kahneman and Treisman, 1992; Becker and Egeth, 2000). In this case, we would expect effects to co-occur and for their strength to be modulated by the SOA. Either of these possible scenarios will provide useful information about the interaction of space- and object-based attention.

## METHOD

### PARTICIPANTS

A total of 174 undergraduate students (63 male) at The George Washington University participated for extra credit in Psychology courses. Twenty participants were placed in SOA 200, 36 in SOA 400, 19 in SOA 700, 38 in SOA 900, 26 in SOA 1200, 35 in SOA 1500. All reported normal or corrected-to-normal visual acuity, and were naïve as to the purpose of the experiment, which was conducted in compliance with the Institutional Review Board (IRB) at the George Washington University.

### APPARATUS

The experiment was completed on a Dell Optiplex 745 computer with a 19-in. screen and a resolution of 1280 × 1024 pixels. Participants were positioned 60 cm away from the screen. The display consisted of one green rectangle and one yellow rectangle set against a black background with a white fixation cross centered between the two rectangles. The fixation cross was 0.4° × 4° of visual angle. The rectangles were 4.2° × 1.2° with 1.8° of visual angle between, and the two together formed a perfect square. The cue, a local thickening of the object outline in red or blue, was 0.2° thick. The target letters, T and L, were 0.7° by 0.33°. The distractors in the other three locations consisted of two target letters superimposed on one another, resulting in a T/L hybrid. The targets and distractors were presented upright (0°), 90°, 180°, or 270°.

### DESIGN AND PROCEDURE

**FIGURE 2:**
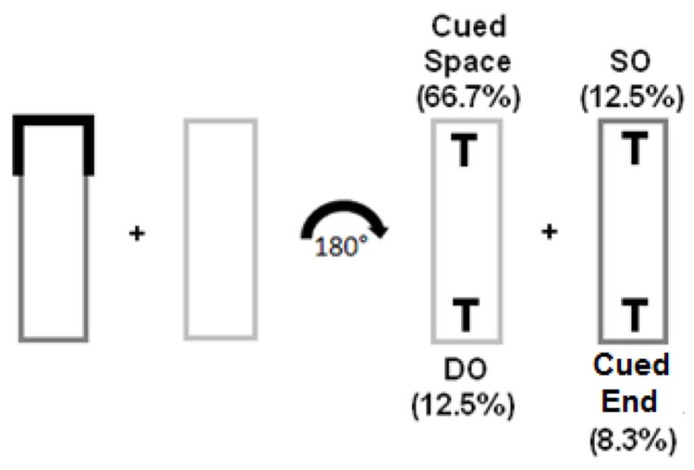
**Trial types of Experiment 1 – High Uncertainty condition.** One rectangle was green (darker shade of grey in this grey scale depiction) and the other was yellow (light grey). Targets could appear in any of the four locations after a 180° rotation (either clockwise or counter-clockwise). Cued Space indicates the target appeared in the original spatial coordinates of the cue, SO indicates the target appeared in the opposite end of the cued object, DO indicates the target appeared in the non-cued object, and cued end indicates the target appeared in the portion of the object that was originally cued.

The experiment was a 4 (validity: valid space, valid object end, invalid same object, invalid different object) × 6 (SOA: 200, 400, 700, 900, 1200, 1500 ms) factorial design. Object type was varied within-subjects while SOA was a between-subjects variable. Cue location, target rotations, and distractor rotations were randomly selected. Participants completed eight blocks of 96 trials for a total of 768 trials. In half of the trials, the display rotated clockwise and in the other half it was rotated counter-clockwise. Valid trials in which the cue was blue to indicate a target appearance in the same spatial location as the cue occurred 66.7% of the time. Invalid trials, as indicated by the red cue, had three possible target locations: same object (SO – 12.5%) where the target appeared in the non-cued end of the cued object, different object (DO – 12.5%) where the target appeared in the non-cued object equidistant from the cue, and cued end of the object trials (CS – 8.3%) where the target appeared in the *part* of the object that originally contained the cue. and which at the end of the rotation was diagonal from the valid spatial location (see **Figure 2**)^1^. In typical static object-based displays, there is no comparable trial type to cued end of the object trials because of the distance disparity from the spatial cue (diagonal vs. adjacent). However, in this experiment it is the crucial trial type because it contains the information from the spatial cue, yet appears in the farthest spatial location. In addition, it is also the least useful for attentional selection because it is the least likely trial type.

Each trial began with a 1000 ms black display with the fixation cross. The rectangles, with one of the four ends overlapped by a blue or red cue, appeared for 100 ms. Then, over a period ranging from 100 to 1400 ms, objects completed a smooth 180° rotation. When combined with the 100 ms cue presentation, the six SOAs were 200, 400, 700, 900, 1200, and 1500 ms. When the rotation was complete, the target display consisted of a T or L in one of the four ends and distractors in the other three locations. The target display remained on the screen until a response was made. The participant responded by pressing the ‘c’ key for T and the ‘m’ key for L. An incorrect response was followed by a feedback screen for 1500 ms instructing the participant to slow down.

### RESULTS AND DISCUSSION

**FIGURE 3:**
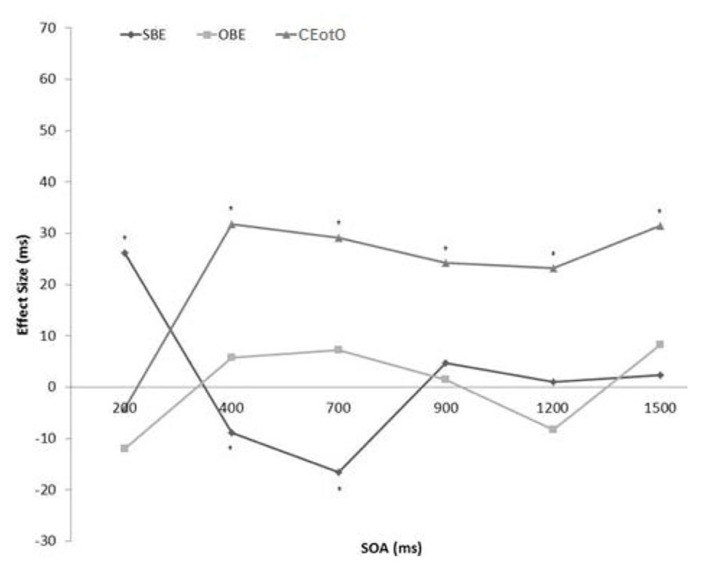
**Effect sizes (ms) of the space-based (valid versus invalid), object-based (SO versus DO), and cued end of the object (cued versus non-cued) comparison in the High Uncertainty condition from SOA 200 to SOA 1500 (significant effects are marked with an asterisk)**.

Only correct responses were analyzed and less than 2% of trials were discarded as a result. There were no significant effects or interactions involving target identity, cue location, absolute target location, rotation direction, or target rotation, so data were collapsed across these variables. For each of the six SOAs, three repeated measures Analyzes of Variance (ANOVAs) were employed to examine: (1) space-based effects (SBEs), by comparing spatially valid targets (blue cues) to spatially invalid targets (red cues) (collapsed over same-object, different-object, and cued end of the object); (2) traditional object-based effects, by comparing same object targets to different object targets; and (3) cued end of the object effects (CEotOs), by comparing targets that appeared at the cued object end (the *part* of the object that was originally cued) to SO and DO targets. **Table 1** lists the reaction times for each of the three analyzes. **Figure 3** plots the three effects across the timecourse: effect size is the difference between valid space and invalid space trials (Space-based), SO and DO trials (Object-based), and cued end of the object and non-cued end of the object trials (Cued End of the Object).

**Table 1:** RTs of each comparison for Experiment 1.

	Space-based effects		Object-based effects		Cued end of the object effects	
SOA (ms)	Valid (ms)	Invalid (ms)	SO (ms)	DO (ms)	Cued (ms)	Non-cued (ms)
200	731.4	705.1	739	727.1	728	723.1
400	748.8	739.9	747.8	753.6	718.4	750
700	732.5	716	721.2	728.4	698.3	727.4
900	743	747.8	756.7	758.3	728.3	752.7
1200	788.4	789.4	802.4	794.2	702.4	725.6
1500	805.7	808.1	816	824.3	783.9	815.3

*Significant effects are marked by shaded cells.*

*Space-based effects.* SOA 200: an ANOVA revealed a significant 26.3 ms effect [*F*(1,19) = 19.508, *p* < 0.001] where valid trials (*M* = 705.1 ms) were faster than invalid trials (*M* = 731.4 ms). SOA 400: a significant -8.9 ms IOR [*F*(1,35) = 4.520, *p* < 0.05] where valid trials (*M* = 748.8 ms) were slower than invalid trials (*M* = 739.9 ms). SOA 700: significant -16.5 ms IOR [*F*(1,18) = 10.503, *p* < 0.01] where valid trials (*M* = 732.5 ms) were slower than invalid trials (*M* = 716 ms). There were no significant effects at the other SOAs.

*Object-based effects (OBEs).* There was no significant effect of object type, such that same-object target identification was not significantly different from different-object target identification, at any of the SOAs.

*Cued End of the Object effects.* SOA 400: an ANOVA revealed a significant 31.6 ms effect [*F*(1,35) = 23.080, *p* < 0.001] where cued end trials (*M* = 718.4 ms) were faster than non-cued end trials (*M* = 750 ms). SOA 700: significant 29.1 ms effect [*F*(1,18) = 18.894, *p* < 0.001] where cued end trials (*M* = 698.3 ms) were faster than non-cued end trials (*M* = 727.4 ms). SOA 900: significant 24.3 ms effect [*F*(1,37) = 9.691, *p* < 0.01] where cued end trials (*M* = 728.3 ms) were faster than non-cued end trials (*M* = 752.7 ms). SOA 1200: significant 23.2 ms effect [*F*(1,25) = 11.241, *p* < 0.01] where cued end trials (*M* = 702.4 ms) were faster than non-cued end trials (*M* = 725.6 ms). SOA 1500, significant 31.4 ms effect [*F*(1,34) = 12.436, *p* < 0.01] where cued end trials (*M* = 783.9 ms) were faster than non-cued end trials (*M* = 815.3 ms). There were no significant effects at SOA 200.

In the current manipulation, the relative contributions of the space- and object-based representations were assessed using a dynamic display to separate the otherwise overlapping representations (Tipper et al., 1994, 1999; Becker and Egeth, 2000; Fallah et al., 2007; List and Robertson, 2007). It was observed that both spatial locations and cued ends of objects guide attention selection. Importantly, however, object-representations did not contribute to attentional guidance as evidenced by the absence of object-based effects. Furthermore, the temporal evolution of these representations was elucidated by manipulating SOA. In this experiment, where the location of the upcoming target was uncertain, it was observed that the space-based representation was prioritized (i.e., guided attention) for 700 ms following the cue onset as evidenced by facilitation at early SOAs and IOR at later SOAs. At the shortest SOA (200 ms) there was a benefit to the cued spatial location, quickly turning into IOR at 400 and persisting until 700 ms. Both the benefit and the IOR indicate that the cued spatial locations were in fact “marked” by attention, strongly supporting the contribution of spatial representation to attentional guidance.

Interestingly, the cued part of the object was tracked throughout the rotation and in most of SOAs, suggesting that the cued end of the object was also prioritized, and that such prioritization is almost immediate and long lasting. This result suggests that the typical benefit observed at the cued location (approximately 100 ms) in studies that utilized a static display was actually a hybrid of the SBE and the CEotO: the spatial location benefits and costs as described previously, and the cued end benefit appeared after 200 ms and persisted at least until 700 ms.

Unexpectedly, and unlike what has been observed in the static display studies, there were no object-based effects at any of the SOAs, which suggest that in dynamic displays only the cued *portion* of the object (i.e., the cued end of the object) receives the benefit of prioritization under conditions of uncertainty, and other locations within an object do not benefit perceptually (i.e., no evidence for attentional spreading). Attention was focused on the cued location and the cued end of the object exclusively and, unlike in static displays, whole objects no longer guided attention.

## EXPERIMENT 2

In Experiment 1, we demonstrated that in the absence of information about the cue-to-target relationship (high degree of uncertainty), cued spatial locations and cued ends of the object both influence attentional selection. The space-based component contributes to attentional guidance at the beginning of the timecourse, but after 900 ms, the sensory signal of the cue dissipates, and selection is determined by the cued end of the object exclusively. Without any target contingencies and after enough processing time was given (>200 ms), attention prioritized the spatial location as well as the cued end of the object, which was tracked throughout the rotation. Therefore, the cue marked both spatial coordinates and the object that occupied them, and the relative contributions changed as the SOA increased.

The purpose of Experiment 2 is to determine the automaticity of the cued end of the object prioritization. Specifically, we asked the following question: if the uncertainty in the input is greatly reduced by introducing predictable contingencies, will the cued end of the object continue to be prioritized or will only spatial factors (e.g., probabilities) guide attentional selection? Rather than equal invalid target probabilities, the red cue was highly predictive of target appearing in a particular spatial location (for half of the subjects appearing in the same-object and in the different-object for the other half), meaning the target would occur there on the majority of trials. If attention is no longer guided by the cued end of the object in the face of local contingencies about the cue-to-target relationship, it will suggest that attentional selection of the cued end of the object is not automatic.

## METHOD

### PARTICIPANTS

Two-hundred and forty-four undergraduate students (78 male) at The George Washington University participated for extra credit in Psychology courses: 35 participants were placed in SOA 200, 36 in SOA 400, 42 in SOA 700, 40 in SOA 900, 43 in SOA 1200, and 48 in SOA 1500. All reported normal or corrected-to-normal visual acuity, and were naïve as to the purpose of the experiment, which was conducted in compliance with the IRB.

### APPARATUS

The apparatus was as described in Experiment 1.

### DESIGN AND PROCEDURE

**FIGURE 4:**
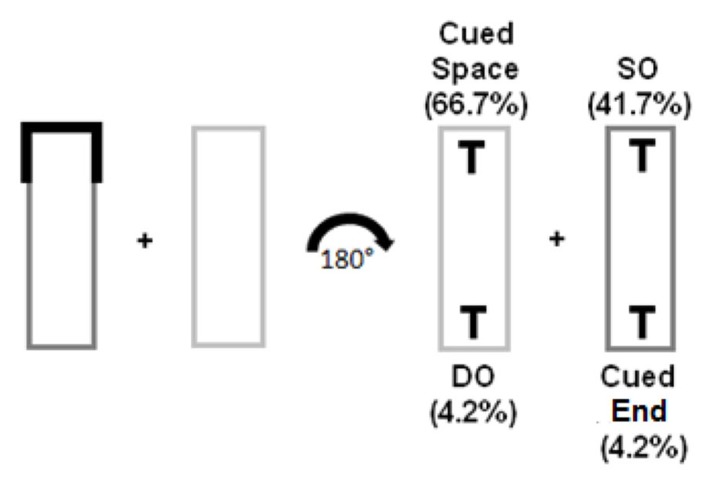
**Trial types of Experiment 2 – low uncertainty condition**.

The design was as described in Experiment 1, with the exception of the distribution of the Invalid trials (red cue trials). Instead of SO, DO, and cued end of the object trials being equally distributed, in this experiment, one object was biased and therefore consisted of the majority of trials. The biased object was a between subject factor: either SO *or *DO was the highly probable target location (41.7% of trials), while the non-biased object and cued end of the object only received 4.3% each (see **Figure 4**).

### RESULTS AND DISCUSSION

**FIGURE 5:**
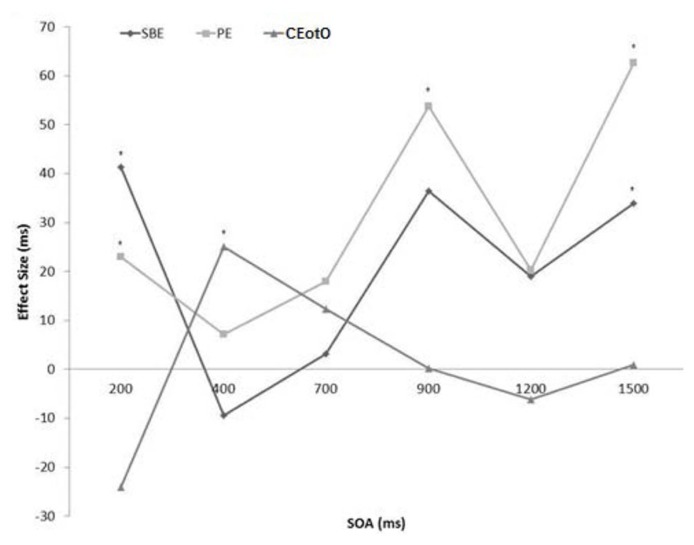
**Effect sizes (ms) of the space-based (valid versus invalid), object-based (SO versus DO), and cued end of the object (cued versus non-cued) comparison in the low uncertainty condition from SOA 200 to SOA 1500 (significant effects are marked with an asterisk)**.

**Table 2** lists the reaction times for each of the three analyzes. **Figure 5** plots the three effects across the timecourse: effect size is the difference between valid space and invalid space trials (Space-based), biased and non-biased trials (Probability), and cued end of the object and non-cued end of the object trials (Cued End of the Object).

**Table 2:** RTs of each comparison for Experiment 2.

	Space-based effects		Probability effects		Cued end of the object effects	
SOA (ms)	Valid (ms)	Invalid (ms)	High (ms)	Low (ms)	Cued (ms)	Non-cued (ms)
200	729.2	770.5	755.1	778.1	778.2	754.1
400	728.9	719.5	728.1	731.2	703	728.1
700	755.1	758.3	754.4	772.3	748.2	760.6
900	779.8	816.2	794	847.8	807	807.2
1200	729.3	748.3	738.1	758.5	748.2	742
1500	772	806	779.2	841.9	796.9	797.7

*
Significant comparisons are marked by shaded cells. Object-based effects are not listed in the table because of a lack of significance at any SOA.*

*Space-based effects.* SOA 200: an ANOVA revealed a significant 41.3 ms effect [*F*(1,36) = 19.378, *p* < 0.001] where Valid trials (*M* = 729.2 ms) were faster than Invalid trials (*M* = 770.5 ms). SOA 1500: significant 34 ms effect [*F*(1,52) = 3.895, *p* = 0.054] where Valid trials (*M* = 772 ms) were faster than Invalid trials (*M* = 806 ms). There were no significant effects at any of the intervening SOAs.

*Probability effects (PEs).* Either SO or DO was biased in terms of higher target probability and an ANOVA revealed no difference in the size of the effect (i.e., no object-based difference), therefore data were collapsed across object type. SOA 200: an ANOVA revealed a significant 23 ms effect [*F*(1,36) = 6.653, *p* < 0.05] where high probability trials (*M* = 755.1 ms) were faster than low probability trials (*M* = 778.1 ms). SOA 900: a significant 53.8 ms effect [*F*(1,33) = 3.413, *p* = 0.072] where high probability trials (*M* = 794 ms) were faster than low probability trials (*M* = 847.8 ms). SOA 1500: a significant 62.7 ms effect [*F*(1,52) = 7.809, *p* < 0.01] where high probability trials (*M* = 779.2 ms) were faster than low probability trials (*M* = 841.9 ms).

*Cued End of the Object effects.* The only significant effect was at SOA 400. There was a significant 25.1 ms effect [*F*(1,34) = 7.940, *p* < 0.05]: where cued end of the object trials (*M* = 703 ms) were faster than non-cued end of the object trials (*M* = 728.1 ms; see **Figure 5**).

Here we investigated whether the CEotO reflects an automatic prioritization of the object surface that occupies the same spatial location as the preceding spatial cue, by introducing an alternative probabilistic strategy, thus reducing uncertainty in the input. With information about where the target is more likely to occur, spatial locations only contributed at 200 and 1500 ms (note the absence of IOR; Muller and Rabbitt, 1989; Wright and Richard, 2000; Lupianez et al., 2001; List and Robertson, 2007). The influence of the cued end of the object was only present at 400 ms SOA, compared to its extensive contribution in Experiment 1, where the location of the upcoming target was uncertain. Probabilities were used to prioritize attentional orienting, which is demonstrated by the PE: high probability locations had faster reaction times than low probability locations at 200 ms, 900 ms, and 1500 ms SOAs. Since the most likely target location could be identified and selected, spatial locations and the cued end of the object no longer exerted an influence. This alternative strategy of prioritizing the biased location contributed to attentional guidance, demonstrating the mechanism’s adaptability and flexibility.

## GENERAL DISCUSSION

When examining factors influencing attentional guidance, it is important to examine not only the contribution of different representations to attentional selection, but also to understand the extent of their contributions over time, and under conditions of varying certainty regarding the visual input. Gaining such fine-grained insight will contribute to a more detailed understanding of attentional guidance and prioritization, by determining its focus, the automaticity of this process based on the information available about the local perceptual contingencies, and how the relationship of costs and benefits changes over time.

In this study, object representations were decoupled from their underlying spatial representations in order to examine individual contributions to attentional selection as a function of time and certainty. We observed that: (i) space- and object-based representations are used for selection concurrently, albeit to varying degrees, (ii) each representation’s strength of contribution changes over time, and (iii) the influence of each representation is not automatic – in other words, it varies depending on the degree of certainty in the visual input. In fact, we demonstrated that the use of space- and object-based representations changes drastically when uncertainty in the visual input is reduced (by introducing probabilistic biasing). In addition to elucidating the relative contribution of each representation under varying conditions (time and certainty), these results provide further evidence for the attentional prioritization hypothesis, which argues for a non-automatic allocation of attentional resources that is based on local contingencies (i.e., the cued location under conditions of uncertainty, or a specific location with some degree of certainty; Shomstein and Yantis, 2002, 2004; Molenberghs et al., 2007; Serences and Yantis, 2007; Chan and Hayward, 2008; Feria, 2008, 2010; Shomstein and Behrmann, 2008; Drummond and Shomstein, 2010; Lee and Shomstein, 2013; Shomstein, 2012; Shomstein and Johnson, 2013).

To summarize the findings, we first observed that with no predictive information about the cue-to-target relationship (i.e., high degree of uncertainty) both spatial locations and surfaces that occupy them guide selection. The space-based representation guided attention until 700 ms following spatial cue (evidenced by facilitation and IOR), at which point the sensory signal of the cue dissipated but the cued end of the object persisted in guiding selection throughout the timecourse (from 400 to 1500 ms). The cued end of the object received the benefit of prioritization, much the same way as the validity or cuing effect has been described in the literature. Attention selected the end of the object in addition to the spatial coordinates underlying the cue, and therefore, after 200 ms SOA there was an observed benefit to the cued end and a cost to the cued spatial location. The relationship between the spatial coordinates and the object is suggestive of over-additive effects: when combined, the two representations produced large significant effects, but when separated, both effects decreased dramatically in size (but not in significance; Saenz et al., 2006; Carrasco, 2011). Once the two representations have been separated, *relevant* information can be selected rather than *all* information, which leads to increased efficiency in selection.

We then observed that when one location was biased in terms of target location probability, attention successfully selected the location where the target was most likely to occur, which led to a benefit for the biased object, regardless of whether it was SO or DO. Rather than relying on the reflexive orienting caused by the cue (in either spatial or object coordinates), attention was guided solely by local contingencies determined by probability imbalances. This effect is consistent with probability based guidance of attention (Muller and Rabbitt, 1989; Geng and Behrmann, 2002; Shomstein and Yantis, 2002, 2004; Feria, 2008). SBEs were only present at the beginning and end of the timecourse (200 and 1500 ms respectively), while the CEotO all but disappeared (only present at 400 ms).

Studies by Tipper et al. (1991, 1994, 1999) have repeatedly demonstrated the separate influences of space- and object-based attention within the context of IOR. Specifically, IOR was discovered in the object-based representation, not only at fixed coordinates (Tipper et al., 1991, 1999). It was suggested that object-based IOR is much more adaptive than space-based, and that attention has a “pursuit” setting for objects rather than solely relying on static spatial locations (Tipper et al., 1991, 1994). We only found IOR for spatial locations, and a benefit for the cued end of the object, which is also strong evidence for a pursuit setting, just one that does not take whole objects into account. When an object is cued and then moves, there is no reason for attention to select the entire object. Instead it can be narrowly focused on the portion of interest and “pursue” it. Dynamic displays might change the nature of attentional selection, such that whole objects are not selected when other information is available. If the motion were to break down or slow down, this could reengage object-based attention as there would be more time for attention to focus on objects.****

IOR has previously been studied over multiple SOAs, and it was found that space- and object-based attention are parallel but separate, and their relationship changes over time (List and Robertson, 2007). Object-based effects as well as space-based IOR were observed in two experiments; however, when the central cue was removed, the object-based effect became “elusive.” We also did not observe object-based effects, but it is also clear that the representations were used concurrently, and that their contributions wax and wane over time. The differences between our findings may come from the amount of rotation accomplished (45° versus our 180°) – it could be due to the perception of motion being less smooth, or perhaps more spatial movement results in object representations being discarded. There is evidence to suggest that prioritization can accompany moving objects (Feria, 2008), which is exactly what we demonstrated with the CEotO. In a set of experiments, there was a bias for the center of an object under conditions of uncertainty. However, with 100% certainty, that bias disappeared in both static and dynamic display. The default setting for attention was to select the center of an object, but when information was introduced about the target location, the target location was selected. This is similar to what we observed in the current experiments (cued end of the object was selected in the absence of information, but the biased object was selected when the bias was obvious) and in a previous study (Drummond and Shomstein, 2010): when an alternative strategy is available, attentional selection can quickly adapt.

The current experiments probed a long timecourse of attentional selection, from 200 to 1500 ms SOA. Contrary to Becker and Egeth (2000), we only found space-based IOR, not object-based IOR. In our experiments there was no spreading of IOR to the rest of the cued object, nor were there any object-based effects. On the surface, it might seem as if our results are inconsistent with those of Becker and Egeth (2000), however there exists a clear explanation as to why we did not observe object-based effects. In the current study, whole objects stopped influencing attention with the introduction of the cued end of the object trial, which does not exist in Becker and Egeth’s study. In the absence of any information from the red cue, the portion of the object that contained the cue was selected by attention, which eliminated the need for object-based attention and therefore any object-based effects. Our results show no IOR when the cue was informative in Experiment 2, similar to previous studies (Posner et al., 1985; Wright and Richard, 2000). Wright and Richard (2000) suggested that there is no reason for inhibition when the target location is known in advance, which leads to an absence of IOR with predictive cues. This is very similar to the *attentional prioritization* hypothesis of Shomstein and Yantis (2002, 2004). Only in the case of target location uncertainty did we find inhibition effects.

These results are also consistent with other theories of attentional guidance, namely with Goldsmith and Yeari’s “window of attention” (2003). In the uncertainty condition, attention was spread across the entire display, which resulted in a large attentional window that encompassed all aspects of the display. In this case, spatial locations and ends of objects were prioritized, resulting in a benefit and then a cost for spatial locations and a consistent benefit for the cued portion of the object. However, when the target location was biased, the attentional window could become narrower to focus on the biased object, which resulted in a benefit for the most likely target location and no object-based differences. Christ et al. (2002) studied IOR with dynamic displays where the cued end of the object moved away from and then returned to the cued spatial location. In this case, very little IOR was observed. These results can be explained by our current results: the cued end of the object was tracked through the movement of the display back to the original location. Under these circumstances, we would expect there to be a benefit – or at least no cost due to a canceling out of spatial IOR and end of object benefit – to the cued location.

Uncertainty plays an important role in attentional selection and the focus of prioritization changes over time. In the presence of target location uncertainty, spatial locations and objects guide attention. When there is a lack of information about the scene, attention is guided by multiple factors and both representations influence selection. As more information is gathered and contingencies are learned, uncertainty is reduced. Prioritization adapts quickly and the new contingencies are used to establish a priority map and become the sole influence on attentional selection. In both cases, more time only solidifies the focus of prioritization: either the cued end of the object or the biased location. Rather than automatically enhancing pre-determined surfaces or locations, attention adapts to the environment and is a dynamic and flexible process. This is further evidence for attentional prioritization and the role of uncertainty reduction in selection.

## Conflict of Interest Statement

The authors declare that the research was conducted in the absence of any commercial or financial relationships that could be construed as a potential conflict of interest.

## Footnotes

1 The difference in percentage from SO and DO – 12.5 vs. 8.3% – amounts to a small difference in trials over the course of the experiment, and was a by-product of SO and DO having the exact same number of trials.
